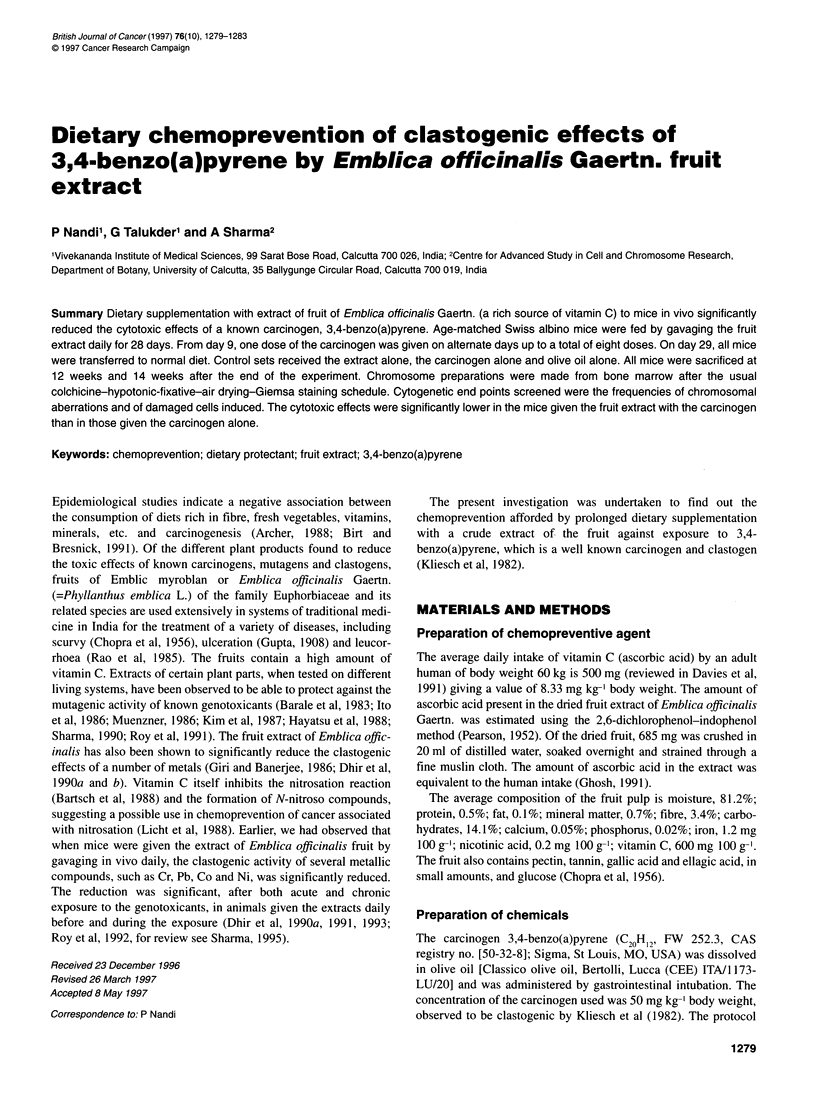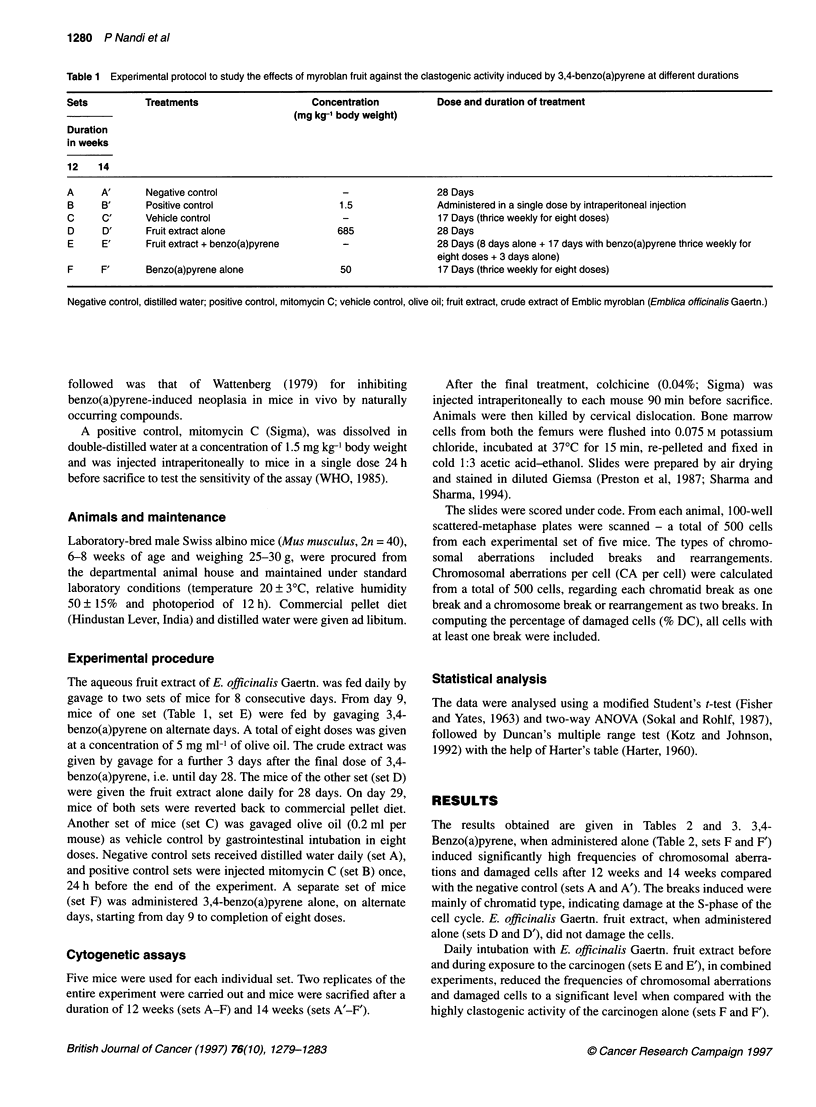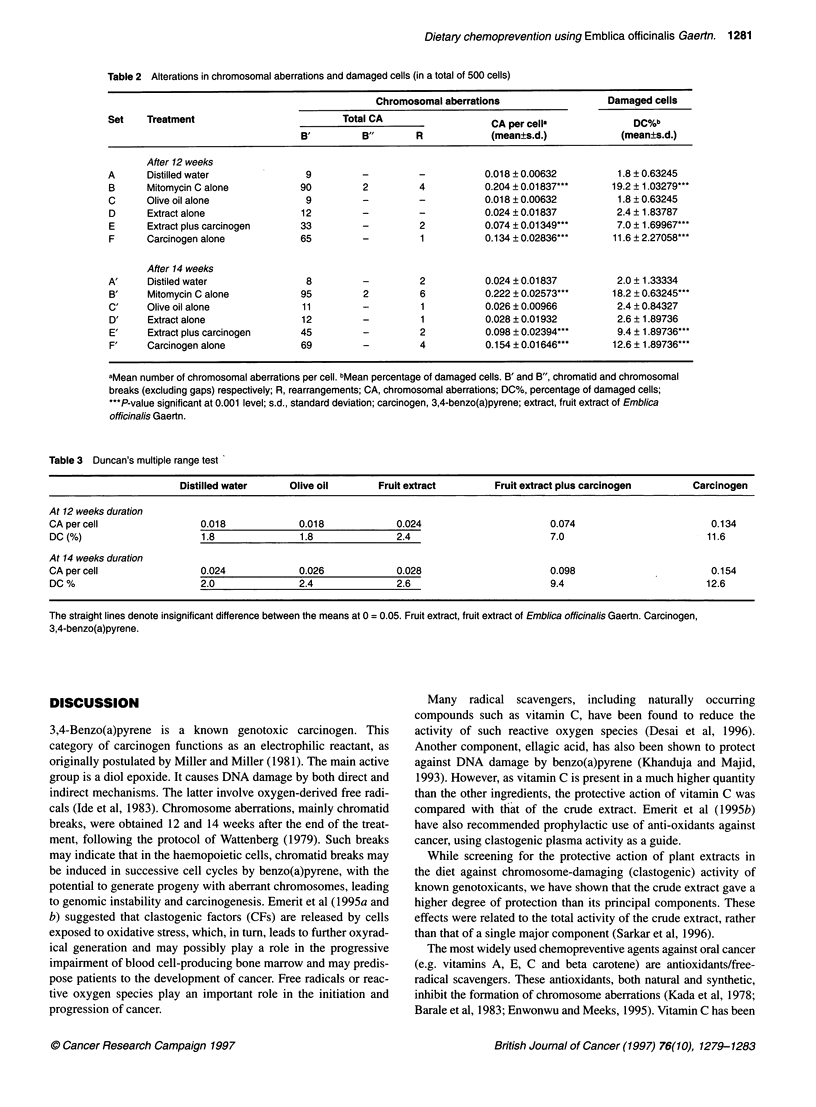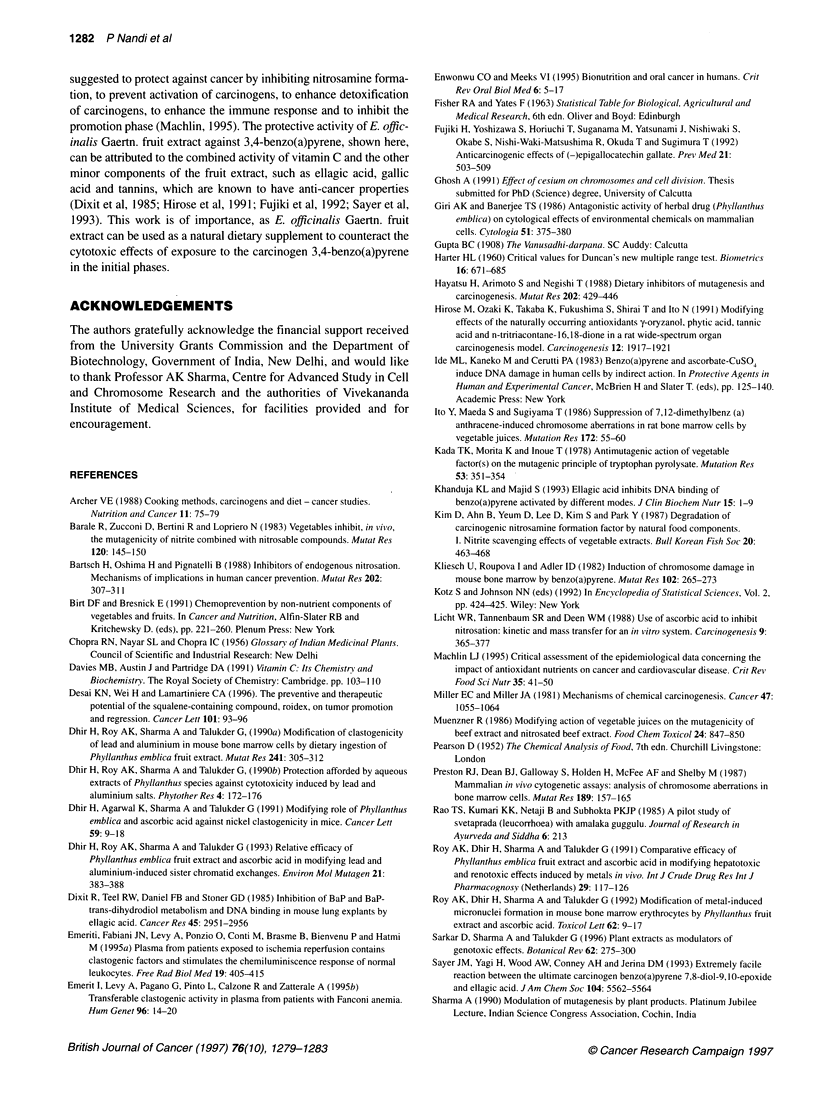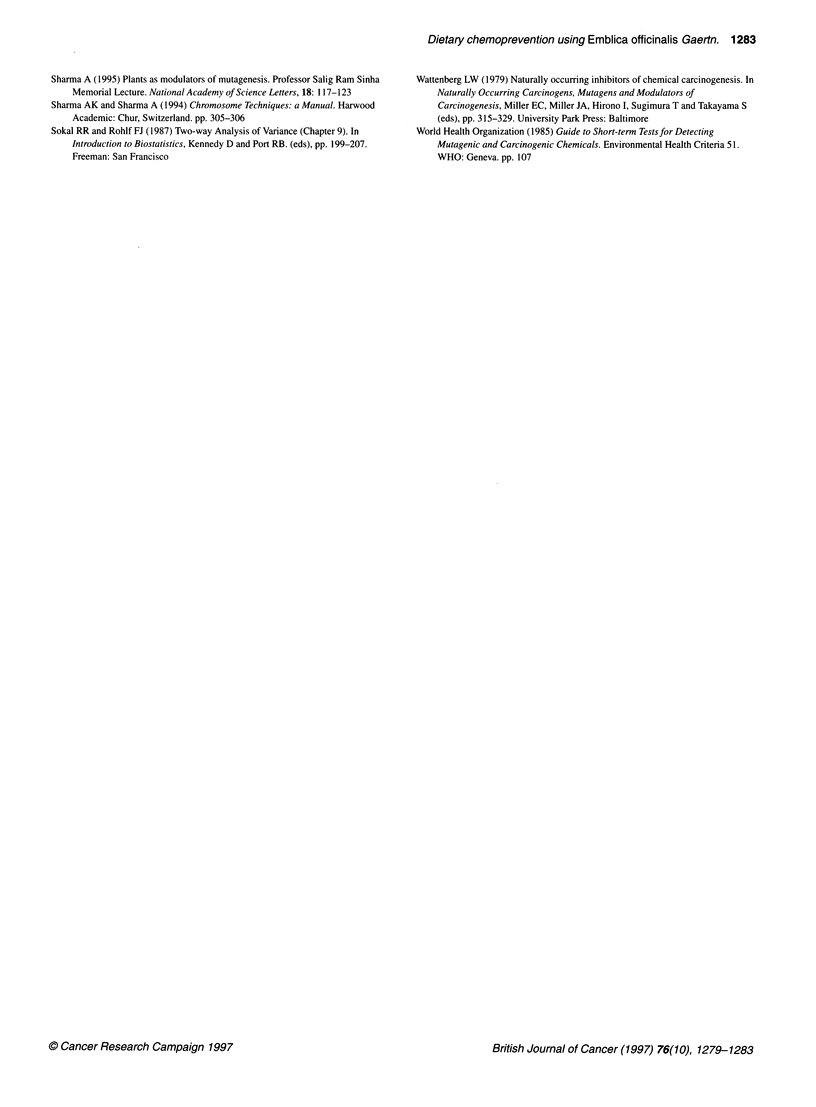# Dietary chemoprevention of clastogenic effects of 3,4-benzo(a)pyrene by Emblica officinalis Gaertn. fruit extract.

**DOI:** 10.1038/bjc.1997.548

**Published:** 1997

**Authors:** P. Nandi, G. Talukder, A. Sharma

**Affiliations:** Vivekananda Institute of Medical Sciences, Calcutta, India.

## Abstract

Dietary supplementation with extract of fruit of Emblica officinalis Gaertn. (a rich source of vitamin C) to mice in vivo significantly reduced the cytotoxic effects of a known carcinogen, 3,4-benzo(a)pyrene. Age-matched Swiss albino mice were fed by gavaging the fruit extract daily for 28 days. From day 9, one dose of the carcinogen was given on alternate days up to a total of eight doses. On day 29, all mice were transferred to normal diet. Control sets received the extract alone, the carcinogen alone and olive oil alone. All mice were sacrificed at 12 weeks and 14 weeks after the end of the experiment. Chromosome preparations were made from bone marrow after the usual colchicine-hypotonic-fixative-air drying-Giemsa staining schedule. Cytogenetic end points screened were the frequencies of chromosomal aberrations and of damaged cells induced. The cytotoxic effects were significantly lower in the mice given the fruit extract with the carcinogen than in those given the carcinogen alone.


British Journal of Cancer (1997) 76(10), 1279–1283
© 1997 Cancer Research Campaign

### Dietary chemoprevention of clastogenic effects of 3,4-benzo(a)pyrene by *Emblica officinalis* Gaertn. fruit extract

**P Nandi[1], G Talukder[1] and A Sharma[2]**

[1]Vivekananda Institute of Medical Sciences, 99 Sarat Bose Road, Calcutta 700 026, India; [2]Centre for Advanced Study in Cell and Chromosome Research, Department of Botany, University of Calcutta, 35 Ballygunge Circular Road, Calcutta 700 019, India

**Summary** Dietary supplementation with extract of fruit of *Emblica officinalis* Gaertn. (a rich source of vitamin C) to mice in vivo significantly reduced the cytotoxic effects of a known carcinogen, 3,4-benzo(a)pyrene. Age-matched Swiss albino mice were fed by gavaging the fruit extract daily for 28 days. From day 9, one dose of the carcinogen was given on alternate days up to a total of eight doses. On day 29, all mice were transferred to normal diet. Control sets received the extract alone, the carcinogen alone and olive oil alone. All mice were sacrificed at 12 weeks and 14 weeks after the end of the experiment. Chromosome preparations were made from bone marrow after the usual colchicine–hypotonic-fixative–air drying–Giemsa staining schedule. Cytogenetic end points screened were the frequencies of chromosomal aberrations and of damaged cells induced. The cytotoxic effects were significantly lower in the mice given the fruit extract with the carcinogen than in those given the carcinogen alone.

**Keywords:** chemoprevention; dietary protectant; fruit extract; 3,4-benzo(a)pyrene

Epidemiological studies indicate a negative association between the consumption of diets rich in fibre, fresh vegetables, vitamins, minerals, etc. and carcinogenesis (Archer, 1988; Birt and Bresnick, 1991). Of the different plant products found to reduce the toxic effects of known carcinogens, mutagens and clastogens, fruits of Emblic myroblan or *Emblica officinalis* Gaertn. (=*Phyllanthus emblica* L.) of the family Euphorbiaceae and its related species are used extensively in systems of traditional medicine in India for the treatment of a variety of diseases, including scurvy (Chopra et al, 1956), ulceration (Gupta, 1908) and leucorrhoea (Rao et al, 1985). The fruits contain a high amount of vitamin C. Extracts of certain plant parts, when tested on different living systems, have been observed to be able to protect against the mutagenic activity of known genotoxicants (Barale et al, 1983; Ito et al, 1986; Muenzner, 1986; Kim et al, 1987; Hayatsu et al, 1988; Sharma, 1990; Roy et al, 1991). The fruit extract of *Emblica officinalis* has also been shown to significantly reduce the clastogenic effects of a number of metals (Giri and Banerjee, 1986; Dhir et al, 1990*a* and *b*). Vitamin C itself inhibits the nitrosation reaction (Bartsch et al, 1988) and the formation of *N*-nitroso compounds, suggesting a possible use in chemoprevention of cancer associated with nitrosation (Licht et al, 1988). Earlier, we had observed that when mice were given the extract of *Emblica officinalis* fruit by gavaging in vivo daily, the clastogenic activity of several metallic compounds, such as Cr, Pb, Co and Ni, was significantly reduced. The reduction was significant, after both acute and chronic exposure to the genotoxicants, in animals given the extracts daily before and during the exposure (Dhir et al, 1990*a*, 1991, 1993; Roy et al, 1992, for review see Sharma, 1995).

*Received 23 December 1996*
*Revised 26 March 1997*
*Accepted 8 May 1997*

*Correspondence to:* P Nandi

The present investigation was undertaken to find out the chemoprevention afforded by prolonged dietary supplementation with a crude extract of the fruit against exposure to 3,4-benzo(a)pyrene, which is a well known carcinogen and clastogen (Kliesch et al, 1982).

### MATERIALS AND METHODS

### Preparation of chemopreventive agent

The average daily intake of vitamin C (ascorbic acid) by an adult human of body weight 60 kg is 500 mg (reviewed in Davies et al, 1991) giving a value of 8.33 mg $kg^{-1}$ body weight. The amount of ascorbic acid present in the dried fruit extract of *Emblica officinalis* Gaertn. was estimated using the 2,6-dichlorophenol–indophenol method (Pearson, 1952). Of the dried fruit, 685 mg was crushed in 20 ml of distilled water, soaked overnight and strained through a fine muslin cloth. The amount of ascorbic acid in the extract was equivalent to the human intake (Ghosh, 1991).

The average composition of the fruit pulp is moisture, 81.2%; protein, 0.5%; fat, 0.1%; mineral matter, 0.7%; fibre, 3.4%; carbohydrates, 14.1%; calcium, 0.05%; phosphorus, 0.02%; iron, 1.2 mg 100 $g^{-1}$; nicotinic acid, 0.2 mg 100 $g^{-1}$; vitamin C, 600 mg 100 $g^{-1}$. The fruit also contains pectin, tannin, gallic acid and ellagic acid, in small amounts, and glucose (Chopra et al, 1956).

### Preparation of chemicals

The carcinogen 3,4-benzo(a)pyrene ($C_{20}H_{12}$, FW 252.3, CAS registry no. [50-32-8]; Sigma, St Louis, MO, USA) was dissolved in olive oil [Classico olive oil, Bertolli, Lucca (CEE) ITA/1173-LU/20] and was administered by gastrointestinal intubation. The concentration of the carcinogen used was 50 mg $kg^{-1}$ body weight, observed to be clastogenic by Kliesch et al (1982). The protocol

Table 1 Experimental protocol to study the effects of myroblan fruit against the clastogenic activity induced by 3,4-benzo(a)pyrene at different durations

| Sets: Duration in weeks 12 | 14 | Treatments | Concentration ($mg\ kg^{-1}$ body weight) | Dose and duration of treatment |
|---|---|---|---|---|
| A | A′ | Negative control | – | 28 Days |
| B | B′ | Positive control | 1.5 | Administered in a single dose by intraperitoneal injection |
| C | C′ | Vehicle control | – | 17 Days (thrice weekly for eight doses) |
| D | D′ | Fruit extract alone | 685 | 28 Days |
| E | E′ | Fruit extract + benzo(a)pyrene | – | 28 Days (8 days alone + 17 days with benzo(a)pyrene thrice weekly for eight doses + 3 days alone) |
| F | F′ | Benzo(a)pyrene alone | 50 | 17 Days (thrice weekly for eight doses) |

Negative control, distilled water; positive control, mitomycin C; vehicle control, olive oil; fruit extract, crude extract of Emblic myroblan (*Emblica officinalis* Gaertn.)

followed was that of Wattenberg (1979) for inhibiting benzo(a)pyrene-induced neoplasia in mice in vivo by naturally occurring compounds.

A positive control, mitomycin C (Sigma), was dissolved in double-distilled water at a concentration of 1.5 mg $kg^{-1}$ body weight and was injected intraperitoneally to mice in a single dose 24 h before sacrifice to test the sensitivity of the assay (WHO, 1985).

### Animals and maintenance

Laboratory-bred male Swiss albino mice (*Mus musculus*, $2n = 40$), 6–8 weeks of age and weighing 25–30 g, were procured from the departmental animal house and maintained under standard laboratory conditions (temperature $20 \pm 3$°C, relative humidity $50 \pm 15$% and photoperiod of 12 h). Commercial pellet diet (Hindustan Lever, India) and distilled water were given ad libitum.

### Experimental procedure

The aqueous fruit extract of *E. officinalis* Gaertn. was fed daily by gavage to two sets of mice for 8 consecutive days. From day 9, mice of one set (Table 1, set E) were fed by gavaging 3,4-benzo(a)pyrene on alternate days. A total of eight doses was given at a concentration of 5 mg $ml^{-1}$ of olive oil. The crude extract was given by gavage for a further 3 days after the final dose of 3,4-benzo(a)pyrene, i.e. until day 28. The mice of the other set (set D) were given the fruit extract alone daily for 28 days. On day 29, mice of both sets were reverted back to commercial pellet diet. Another set of mice (set C) was gavaged olive oil (0.2 ml per mouse) as vehicle control by gastrointestinal intubation in eight doses. Negative control sets received distilled water daily (set A), and positive control sets were injected mitomycin C (set B) once, 24 h before the end of the experiment. A separate set of mice (set F) was administered 3,4-benzo(a)pyrene alone, on alternate days, starting from day 9 to completion of eight doses.

### Cytogenetic assays

Five mice were used for each individual set. Two replicates of the entire experiment were carried out and mice were sacrified after a duration of 12 weeks (sets A–F) and 14 weeks (sets A′–F′).

After the final treatment, colchicine (0.04%; Sigma) was injected intraperitoneally to each mouse 90 min before sacrifice. Animals were then killed by cervical dislocation. Bone marrow cells from both the femurs were flushed into 0.075 M potassium chloride, incubated at 37°C for 15 min, re-pelleted and fixed in cold 1:3 acetic acid–ethanol. Slides were prepared by air drying and stained in diluted Giemsa (Preston et al, 1987; Sharma and Sharma, 1994).

The slides were scored under code. From each animal, 100-well scattered-metaphase plates were scanned – a total of 500 cells from each experimental set of five mice. The types of chromosomal aberrations included breaks and rearrangements. Chromosomal aberrations per cell (CA per cell) were calculated from a total of 500 cells, regarding each chromatid break as one break and a chromosome break or rearrangement as two breaks. In computing the percentage of damaged cells (% DC), all cells with at least one break were included.

### Statistical analysis

The data were analysed using a modified Student's *t*-test (Fisher and Yates, 1963) and two-way ANOVA (Sokal and Rohlf, 1987), followed by Duncan's multiple range test (Kotz and Johnson, 1992) with the help of Harter's table (Harter, 1960).

### RESULTS

The results obtained are given in Tables 2 and 3. 3,4-Benzo(a)pyrene, when administered alone (Table 2, sets F and F′) induced significantly high frequencies of chromosomal aberrations and damaged cells after 12 weeks and 14 weeks compared with the negative control (sets A and A′). The breaks induced were mainly of chromatid type, indicating damage at the S-phase of the cell cycle. *E. officinalis* Gaertn. fruit extract, when administered alone (sets D and D′), did not damage the cells.

Daily intubation with *E. officinalis* Gaertn. fruit extract before and during exposure to the carcinogen (sets E and E′), in combined experiments, reduced the frequencies of chromosomal aberrations and damaged cells to a significant level when compared with the highly clastogenic activity of the carcinogen alone (sets F and F′).

© Cancer Research Campaign 1997

**Table 2** Alterations in chromosomal aberrations and damaged cells (in a total of 500 cells)

| Set | Treatment | Chromosomal aberrations | | | | Damaged cells |
|---|---|---|---|---|---|---|
| | | Total CA | | | CA per cell[a] (mean±s.d.) | DC%[b] (mean±s.d.) |
| | | B′ | B″ | R | | |
| | *After 12 weeks* | | | | | |
| A | Distilled water | 9 | – | – | 0.018 ± 0.00632 | 1.8 ± 0.63245 |
| B | Mitomycin C alone | 90 | 2 | 4 | 0.204 ± 0.01837*** | 19.2 ± 1.03279*** |
| C | Olive oil alone | 9 | – | – | 0.018 ± 0.00632 | 1.8 ± 0.63245 |
| D | Extract alone | 12 | – | – | 0.024 ± 0.01837 | 2.4 ± 1.83787 |
| E | Extract plus carcinogen | 33 | – | 2 | 0.074 ± 0.01349*** | 7.0 ± 1.69967*** |
| F | Carcinogen alone | 65 | – | 1 | 0.134 ± 0.02836*** | 11.6 ± 2.27058*** |
| | *After 14 weeks* | | | | | |
| A′ | Distiled water | 8 | – | 2 | 0.024 ± 0.01837 | 2.0 ± 1.33334 |
| B′ | Mitomycin C alone | 95 | 2 | 6 | 0.222 ± 0.02573*** | 18.2 ± 0.63245*** |
| C′ | Olive oil alone | 11 | – | 1 | 0.026 ± 0.00966 | 2.4 ± 0.84327 |
| D′ | Extract alone | 12 | – | 1 | 0.028 ± 0.01932 | 2.6 ± 1.89736 |
| E′ | Extract plus carcinogen | 45 | – | 2 | 0.098 ± 0.02394*** | 9.4 ± 1.89736*** |
| F′ | Carcinogen alone | 69 | – | 4 | 0.154 ± 0.01646*** | 12.6 ± 1.89736*** |

[a]Mean number of chromosomal aberrations per cell. [b]Mean percentage of damaged cells. B′ and B″, chromatid and chromosomal breaks (excluding gaps) respectively; R, rearrangements; CA, chromosomal aberrations; DC%, percentage of damaged cells; ****P*-value significant at 0.001 level; s.d., standard deviation; carcinogen, 3,4-benzo(a)pyrene; extract, fruit extract of *Emblica officinalis* Gaertn.

**Table 3** Duncan's multiple range test

| | Distilled water | Olive oil | Fruit extract | Fruit extract plus carcinogen | Carcinogen |
|---|---|---|---|---|---|
| *At 12 weeks duration* | | | | | |
| CA per cell | 0.018 | 0.018 | 0.024 | 0.074 | 0.134 |
| DC (%) | 1.8 | 1.8 | 2.4 | 7.0 | 11.6 |
| *At 14 weeks duration* | | | | | |
| CA per cell | 0.024 | 0.026 | 0.028 | 0.098 | 0.154 |
| DC % | 2.0 | 2.4 | 2.6 | 9.4 | 12.6 |

The straight lines denote insignificant difference between the means at 0 = 0.05. Fruit extract, fruit extract of *Emblica officinalis* Gaertn. Carcinogen, 3,4-benzo(a)pyrene.

### DISCUSSION

3,4-Benzo(a)pyrene is a known genotoxic carcinogen. This category of carcinogen functions as an electrophilic reactant, as originally postulated by Miller and Miller (1981). The main active group is a diol epoxide. It causes DNA damage by both direct and indirect mechanisms. The latter involve oxygen-derived free radicals (Ide et al, 1983). Chromosome aberrations, mainly chromatid breaks, were obtained 12 and 14 weeks after the end of the treatment, following the protocol of Wattenberg (1979). Such breaks may indicate that in the haemopoietic cells, chromatid breaks may be induced in successive cell cycles by benzo(a)pyrene, with the potential to generate progeny with aberrant chromosomes, leading to genomic instability and carcinogenesis. Emerit et al (1995*a* and *b*) suggested that clastogenic factors (CFs) are released by cells exposed to oxidative stress, which, in turn, leads to further oxyradical generation and may possibly play a role in the progressive impairment of blood cell-producing bone marrow and may predispose patients to the development of cancer. Free radicals or reactive oxygen species play an important role in the initiation and progression of cancer.

Many radical scavengers, including naturally occurring compounds such as vitamin C, have been found to reduce the activity of such reactive oxygen species (Desai et al, 1996). Another component, ellagic acid, has also been shown to protect against DNA damage by benzo(a)pyrene (Khanduja and Majid, 1993). However, as vitamin C is present in a much higher quantity than the other ingredients, the protective action of vitamin C was compared with that of the crude extract. Emerit et al (1995*b*) have also recommended prophylactic use of anti-oxidants against cancer, using clastogenic plasma activity as a guide.

While screening for the protective action of plant extracts in the diet against chromosome-damaging (clastogenic) activity of known genotoxicants, we have shown that the crude extract gave a higher degree of protection than its principal components. These effects were related to the total activity of the crude extract, rather than that of a single major component (Sarkar et al, 1996).

The most widely used chemopreventive agents against oral cancer (e.g. vitamins A, E, C and beta carotene) are antioxidants/free-radical scavengers. These antioxidants, both natural and synthetic, inhibit the formation of chromosome aberrations (Kada et al, 1978; Barale et al, 1983; Enwonwu and Meeks, 1995). Vitamin C has been

*© Cancer Research Campaign 1997*

suggested to protect against cancer by inhibiting nitrosamine formation, to prevent activation of carcinogens, to enhance detoxification of carcinogens, to enhance the immune response and to inhibit the promotion phase (Machlin, 1995). The protective activity of *E. officinalis* Gaertn. fruit extract against 3,4-benzo(a)pyrene, shown here, can be attributed to the combined activity of vitamin C and the other minor components of the fruit extract, such as ellagic acid, gallic acid and tannins, which are known to have anti-cancer properties (Dixit et al, 1985; Hirose et al, 1991; Fujiki et al, 1992; Sayer et al, 1993). This work is of importance, as *E. officinalis* Gaertn. fruit extract can be used as a natural dietary supplement to counteract the cytotoxic effects of exposure to the carcinogen 3,4-benzo(a)pyrene in the initial phases.

### ACKNOWLEDGEMENTS

The authors gratefully acknowledge the financial support received from the University Grants Commission and the Department of Biotechnology, Government of India, New Delhi, and would like to thank Professor AK Sharma, Centre for Advanced Study in Cell and Chromosome Research and the authorities of Vivekananda Institute of Medical Sciences, for facilities provided and for encouragement.

### REFERENCES

Archer VE (1988) Cooking methods, carcinogens and diet – cancer studies. *Nutrition and Cancer* **11**: 75–79

Barale R, Zucconi D, Bertini R and Lopriero N (1983) Vegetables inhibit, *in vivo*, the mutagenicity of nitrite combined with nitrosable compounds. *Mutat Res* **120**: 145–150

Bartsch H, Oshima H and Pignatelli B (1988) Inhibitors of endogenous nitrosation. Mechanisms of implications in human cancer prevention. *Mutat Res* **202**: 307–311

Birt DF and Bresnick E (1991) Chemoprevention by non-nutrient components of vegetables and fruits. In *Cancer and Nutrition*, Alfin-Slater RB and Kritchewsky D. (eds), pp. 221–260. Plenum Press: New York

Chopra RN, Nayar SL and Chopra IC (1956) *Glossary of Indian Medicinal Plants*. Council of Scientific and Industrial Research: New Delhi

Davies MB, Austin J and Partridge DA (1991) *Vitamin C: Its Chemistry and Biochemistry*. The Royal Society of Chemistry: Cambridge. pp. 103–110

Desai KN, Wei H and Lamartiniere CA (1996). The preventive and therapeutic potential of the squalene-containing compound, roidex, on tumor promotion and regression. *Cancer Lett* **101**: 93–96

Dhir H, Roy AK, Sharma A and Talukder G, (1990*a*) Modification of clastogenicity of lead and aluminium in mouse bone marrow cells by dietary ingestion of *Phyllanthus emblica* fruit extract. *Mutat Res* **241**: 305–312

Dhir H, Roy AK, Sharma A and Talukder G, (1990*b*) Protection afforded by aqueous extracts of *Phyllanthus* species against cytotoxicity induced by lead and aluminium salts. *Phytother Res* **4**: 172–176

Dhir H, Agarwal K, Sharma A and Talukder G (1991) Modifying role of *Phyllanthus emblica* and ascorbic acid against nickel clastogenicity in mice. *Cancer Lett* **59**: 9–18

Dhir H, Roy AK, Sharma A and Talukder G (1993) Relative efficacy of *Phyllanthus emblica* fruit extract and ascorbic acid in modifying lead and aluminium-induced sister chromatid exchanges. *Environ Mol Mutagen* **21**: 383–388

Dixit R, Teel RW, Daniel FB and Stoner GD (1985) Inhibition of BaP and BaP-trans-dihydrodiol metabolism and DNA binding in mouse lung explants by ellagic acid. *Cancer Res* **45**: 2951–2956

Emeriti, Fabiani JN, Levy A, Ponzio O, Conti M, Brasme B, Bienvenu P and Hatmi M (1995*a*) Plasma from patients exposed to ischemia reperfusion contains clastogenic factors and stimulates the chemiluminiscence response of normal leukocytes. *Free Rad Biol Med* **19**: 405–415

Emerit I, Levy A, Pagano G, Pinto L, Calzone R and Zatterale A (1995*b*) Transferable clastogenic activity in plasma from patients with Fanconi anemia. *Hum Genet* **96**: 14–20

Enwonwu CO and Meeks VI (1995) Bionutrition and oral cancer in humans. *Crit Rev Oral Biol Med* **6**: 5–17

Fisher RA and Yates F (1963) *Statistical Table for Biological, Agricultural and Medical Research*, 6th edn. Oliver and Boyd: Edinburgh

Fujiki H, Yoshizawa S, Horiuchi T, Suganama M, Yatsunami J, Nishiwaki S, Okabe S, Nishi-Waki-Matsushima R, Okuda T and Sugimura T (1992) Anticarcinogenic effects of (–)epigallocatechin gallate. *Prev Med* **21**: 503–509

Ghosh A (1991) *Effect of cesium on chromosomes and cell division*. Thesis submitted for PhD (Science) degree, University of Calcutta

Giri AK and Banerjee TS (1986) Antagonistic activity of herbal drug (*Phyllanthus emblica*) on cytological effects of environmental chemicals on mammalian cells. *Cytologia* **51**: 375–380

Gupta BC (1908) *The Vanusadhi-darpana*. SC Auddy: Calcutta

Harter HL (1960) Critical values for Duncan's new multiple range test. *Biometrics* **16**: 671–685

Hayatsu H, Arimoto S and Negishi T (1988) Dietary inhibitors of mutagenesis and carcinogenesis. *Mutat Res* **202**: 429–446

Hirose M, Ozaki K, Takaba K, Fukushima S, Shirai T and Ito N (1991) Modifying effects of the naturally occurring antioxidants γ-oryzanol, phytic acid, tannic acid and n-tritriacontane-16,18-dione in a rat wide-spectrum organ carcinogenesis model. *Carcinogenesis* **12**: 1917–1921

Ide ML, Kaneko M and Cerutti PA (1983) Benzo(a)pyrene and ascorbate-$CuSO_4$ induce DNA damage in human cells by indirect action. In *Protective Agents in Human and Experimental Cancer*, McBrien H and Slater T. (eds), pp. 125–140. Academic Press: New York

Ito Y, Maeda S and Sugiyama T (1986) Suppression of 7,12-dimethylbenz (a) anthracene-induced chromosome aberrations in rat bone marrow cells by vegetable juices. *Mutation Res* **172**: 55–60

Kada TK, Morita K and Inoue T (1978) Antimutagenic action of vegetable factor(s) on the mutagenic principle of tryptophan pyrolysate. *Mutation Res* **53**: 351–354

Khanduja KL and Majid S (1993) Ellagic acid inhibits DNA binding of benzo(a)pyrene activated by different modes. *J Clin Biochem Nutr* **15**: 1–9

Kim D, Ahn B, Yeum D, Lee D, Kim S and Park Y (1987) Degradation of carcinogenic nitrosamine formation factor by natural food components. I. Nitrite scavenging effects of vegetable extracts. *Bull Korean Fish Soc* **20**: 463–468

Kliesch U, Roupova I and Adler ID (1982) Induction of chromosome damage in mouse bone marrow by benzo(a)pyrene. *Mutat Res* **102**: 265–273

Kotz S and Johnson NN (eds) (1992) In *Encyclopedia of Statistical Sciences*, Vol. 2, pp. 424–425. Wiley: New York

Licht WR, Tannenbaum SR and Deen WM (1988) Use of ascorbic acid to inhibit nitrosation: kinetic and mass transfer for an *in vitro* system. *Carcinogenesis* **9**: 365–377

Machlin LJ (1995) Critical assessment of the epidemiological data concerning the impact of antioxidant nutrients on cancer and cardiovascular disease. *Crit Rev Food Sci Nutr* **35**: 41–50

Miller EC and Miller JA (1981) Mechanisms of chemical carcinogenesis. *Cancer* **47**: 1055–1064

Muenzner R (1986) Modifying action of vegetable juices on the mutagenicity of beef extract and nitrosated beef extract. *Food Chem Toxicol* **24**: 847–850

Pearson D (1952) *The Chemical Analysis of Food*, 7th edn. Churchill Livingstone: London

Preston RJ, Dean BJ, Galloway S, Holden H, McFee AF and Shelby M (1987) Mammalian *in vivo* cytogenetic assays: analysis of chromosome aberrations in bone marrow cells. *Mutat Res* **189**: 157–165

Rao TS, Kumari KK, Netaji B and Subhokta PKJP (1985) A pilot study of svetaprada (leucorrhoea) with amalaka guggulu. *Journal of Research in Ayurveda and Siddha* **6**: 213

Roy AK, Dhir H, Sharma A and Talukder G (1991) Comparative efficacy of *Phyllanthus emblica* fruit extract and ascorbic acid in modifying hepatotoxic and renotoxic effects induced by metals *in vivo*. *Int J Crude Drug Res Int J Pharmacognosy* (Netherlands) **29**: 117–126

Roy AK, Dhir H, Sharma A and Talukder G (1992) Modification of metal-induced micronuclei formation in mouse bone marrow erythrocytes by *Phyllanthus* fruit extract and ascorbic acid. *Toxicol Lett* **62**: 9–17

Sarkar D, Sharma A and Talukder G (1996) Plant extracts as modulators of genotoxic effects. *Botanical Rev* **62**: 275–300

Sayer JM, Yagi H, Wood AW, Conney AH and Jerina DM (1993) Extremely facile reaction between the ultimate carcinogen benzo(a)pyrene 7,8-diol-9,10-epoxide and ellagic acid. *J Am Chem Soc* **104**: 5562–5564

Sharma A (1990) Modulation of mutagenesis by plant products. Platinum Jubilee Lecture, Indian Science Congress Association, Cochin, India

© Cancer Research Campaign 1997

Sharma A (1995) Plants as modulators of mutagenesis. Professor Salig Ram Sinha Memorial Lecture. *National Academy of Science Letters*, **18**: 117–123

Sharma AK and Sharma A (1994) *Chromosome Techniques: a Manual*. Harwood Academic: Chur, Switzerland. pp. 305–306

Sokal RR and Rohlf FJ (1987) Two-way Analysis of Variance (Chapter 9). In *Introduction to Biostatistics*, Kennedy D and Port RB. (eds), pp. 199–207. Freeman: San Francisco

Wattenberg LW (1979) Naturally occurring inhibitors of chemical carcinogenesis. In *Naturally Occurring Carcinogens, Mutagens and Modulators of Carcinogenesis*, Miller EC, Miller JA, Hirono I, Sugimura T and Takayama S (eds), pp. 315–329. University Park Press: Baltimore

World Health Organization (1985) *Guide to Short-term Tests for Detecting Mutagenic and Carcinogenic Chemicals*. Environmental Health Criteria 51. WHO: Geneva. pp. 107

© Cancer Research Campaign 1997